# Identification of Gene Networks and Pathways Associated with Guillain-Barré Syndrome

**DOI:** 10.1371/journal.pone.0029506

**Published:** 2012-01-10

**Authors:** Kuo-Hsuan Chang, Tzi-Jung Chuang, Rong-Kuo Lyu, Long-Sun Ro, Yih-Ru Wu, Hong-Shiu Chang, Chin-Chang Huang, Hung-Chou Kuo, Wen-Chuin Hsu, Chun-Che Chu, Chiung-Mei Chen

**Affiliations:** Department of Neurology, Chang Gung Memorial Hospital Linkou Medical Center and College of Medicine, Chang Gung University, Taiwan, Republic of China; City of Hope National Medical Center and Beckman Research Institute, United States of America

## Abstract

**Background:**

The underlying change of gene network expression of Guillain-Barré syndrome (GBS) remains elusive. We sought to identify GBS-associated gene networks and signaling pathways by analyzing the transcriptional profile of leukocytes in the patients with GBS.

**Methods and Findings:**

Quantitative global gene expression microarray analysis of peripheral blood leukocytes was performed on 7 patients with GBS and 7 healthy controls. Gene expression profiles were compared between patients and controls after standardization. The set of genes that significantly correlated with GBS was further analyzed by Ingenuity Pathways Analyses.

256 genes and 18 gene networks were significantly associated with GBS (fold change ≥2, *P*<0.05). *FOS*, *PTGS2*, *HMGB2* and *MMP9* are the top four of 246 significantly up-regulated genes. The most significant disease and altered biological function genes associated with GBS were those involved in inflammatory response, infectious disease, and respiratory disease. Cell death, cellular development and cellular movement were the top significant molecular and cellular functions involved in GBS. Hematological system development and function, immune cell trafficking and organismal survival were the most significant GBS-associated function in physiological development and system category. Several hub genes, such as *MMP9*, *PTGS2* and *CREB1* were identified in the associated gene networks. Canonical pathway analysis showed that GnRH, corticotrophin-releasing hormone and ERK/MAPK signaling were the most significant pathways in the up-regulated gene set in GBS.

**Conclusions:**

This study reveals the gene networks and canonical pathways associated with GBS. These data provide not only networks between the genes for understanding the pathogenic properties of GBS but also map significant pathways for the future development of novel therapeutic strategies.

## Introduction

Guillain-Barré syndrome (GBS) is an inflammatory demyelinating disease of the peripheral nervous system that is characterized by acute areflexic paralysis [1]. As the major cause of acute neuromuscular paralysis around the world, the annual incidence of GBS is 0.62 to 2.66 cases per population of 100,000 [2]. GBS is thought to be an autoimmune disease triggered by antecedent infection [1], [3], [4], [5], [6], [7]. Currently the underlying mechanisms of this immune-mediated invasion of nerves remain elusive. A number of infectious agents, such as Campylobacter jejuni and Mycoplasma, are proposed to induce T cell-mediated immune process against myelin sheath proteins or gangliosides [7], [8], [9], [10], [11], [12], [13]. The activated T cells could induce the production of autoantibodies or recruit macrophages on the surface of myelin sheath or the node of Ranvier [14], [15], [16], [17]. The mediators released by activated macrophages may cause destruction of myelin sheath or axons [18], [19]. Although a number of studies have shown the crucial role of inflammatory infiltration in such demyelination or axonal degeneration [15], [16], [20], [21], [22], the alteration of cellular entity in these inflammatory cells has not been completely revealed.

So far a long list of GBS-associated biomarkers, including myelin basic protein [23], neurofilaments [24], anti-ganglioside antibodies [25], neuron-specific enolase [26], S100B [26], hypocretin-1 [27], cystatin C [28], transthyretin [29], haptoglobin [30], [31], carbonylation of albumin [32], and different cytokines and complement factors [33], [34], [35], has been disclosed. These studies, carried out on body fluid analysis, did not provide critical information on the molecular modifications in the inflammatory cells. Moreover, these studies did not reveal information about the changes of systemic signaling networks associated with GBS. In this study, we address both these questions by analyzing the global quantitative gene expression profile in peripheral blood leukocytes. This examination provides the opportunity for understanding the evolution of cell responses and sheds light on screening novel therapeutic targets for GBS.

## Results

### Leucocyte transcription profile in GBS patients

**Figure 1:**
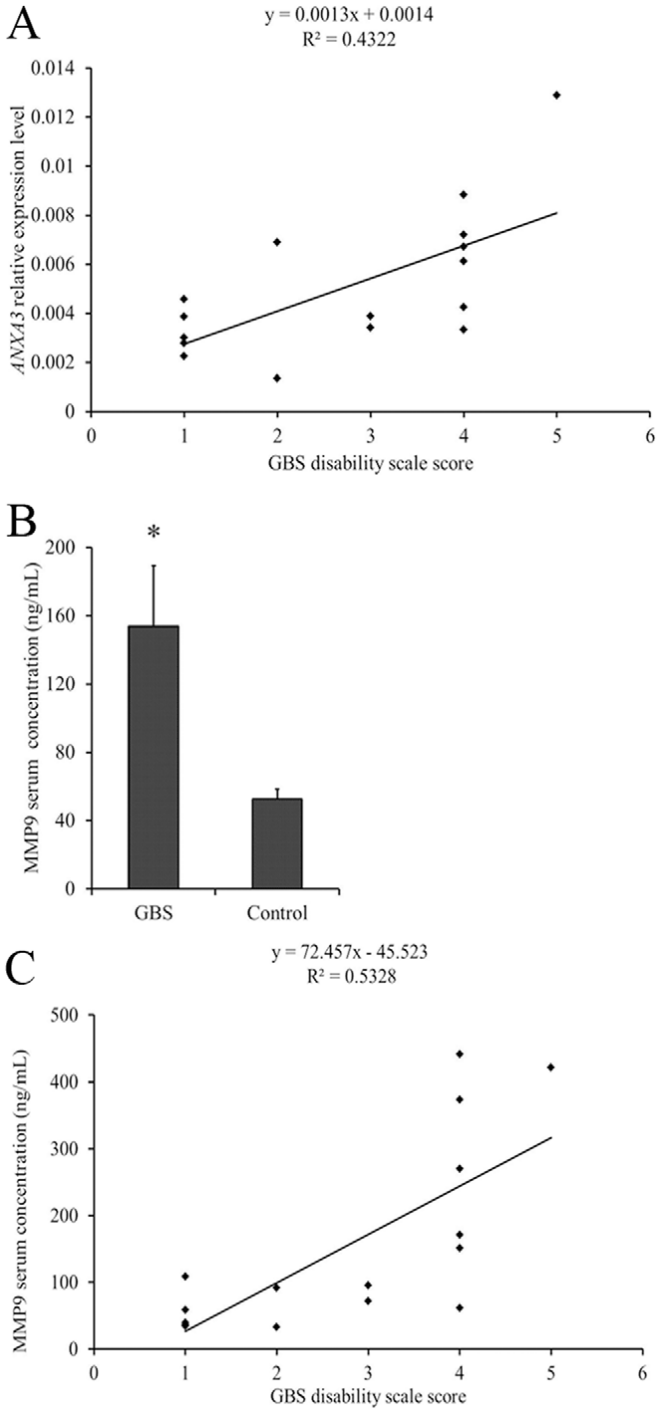
*ANXA3* and *MMP* expression levels in patients with GBS and control. (**A**) Correlation between gene expression of *ANXA3* and GBS disability scale score (*P* = 0.006). (**B**) Serum level of MMP9 in GBS and control groups. (**C**) Correlation between serum level of MMP9 and GBS disability scale score (*P* = 0.001) *Statistically significant in comparison with GBS and control groups (*P* = 0.013). Data are expressed as mean ± standard error.

A total of 2794 transcripts were significantly associated with GBS (*P*<0.05). Of these, 256 genes reached the minimum fold changes (≥2). 246 genes were up-regulated and 10 genes are down-regulated in GBS group, respectively (Table 1 and Table S2). Of 15 genes quantified by RT-PCR, 8 up-regulated genes (*FOS*, *PTGS2*, *HMGB2*, *MMP9*, *LY96*, *TTRAP*, *ANXA3*, *CREB1*) were in good agreement with the results of microarray (Table 2). Furthermore, the *ANXA3* expression level is proportionally correlated with the score of GBS disability scale [36] (Fig. 1A, *P* = 0.006). The GBS group also displayed a significantly higher serum level of MMP9 (Fig. 1B, 153.74±35.68 ng/mL) than the control group (52.70±5.67 ng/mL, *P* = 0.013). The serum level of MMP9 is also positively correlated with GBS disability scale score (Fig. 1C, *P* = 0.001).

**Table 1:** List of the top 20 up-regulated and all down-regulated genes.

Fold change (GBS vs control)	Regulation (GBS vs control)	Gene Symbol
4.022045	up	*FOS*
3.916694	up	*PTGS2*
3.632606	up	*HMGB2*
3.550858	up	*MMP9*
3.432947	up	*DEFA3*
3.272876	up	*LY96*
2.988478	up	*LTF*
2.95113	up	*TTRAP*
2.93675	up	*LILRB2*
2.909457	up	*FPR2*
2.880911	up	*CDC42*
2.876669	up	*IGF2R*
2.832869	up	*NFIL3*
2.814348	up	*SMCHD1*
2.811425	up	*IFRD1*
2.806923	up	*MARCKS*
2.790935	up	*ZNF12*
2.770413	up	*PKN2*
2.764796	up	*KYNU*
2.763209	up	*SENP6*
−4.52657	down	*SELENBP1*
−2.62051	down	*HBQ1*
−2.59983	down	*GSPT1*
−2.50056	down	*PSMF1*
−2.48064	down	*GLRX5*
−2.39151	down	*FBXO7*
−2.13839	down	*HAGH*
−2.12692	down	*GUK1*
−2.01586	down	*NUDT4*
−2.01346	down	*PTGDS*

**Table 2:** Summarized RT-PCR confirmation results of the 15 genes.

Genes	Description	Fold change by arrays (GBS vs Control)	*P* value	Fold changes by RT-PCR (GBS vs Control)	*P* value
*FOS*	FBJ murine osteosarcoma viral oncogene homolog	4.02	0.042	3.78	4.54E-05
*PTGS2*	Prostaglandin-endoperoxide synthase 2	3.92	0.036	3.03	0.011
*HMGB2*	High mobility group box 2	3.63	0.011	3.36	0.019
*MMP9*	Matrix metallopeptidase 9	3.55	8.85E-04	3.22	0.021
*DEFA3*	Defensin, alpha 3, neutrophil-specific	3.43	0.026	1.68	0.263
*LY96*	Lymphocyte antigen 96	3.27	0.037	2.29	1.69E-04
*LTF*	Lactotransferrin	2.99	0.010	1.46	0.42
*TTRAP*	TRAF and TNF receptor-associated protein	2.95	0.023	3.66	0.001
*CREBBP*	CREB binding protein	2.56	0.034	1.02	0.87
*ANXA3*	Annexin A3	2.14	0.017	1.76	0.028
*CASP1*	Caspase 1	2.04	0.038	−1.12	0.301
*CREB1*	cAMP responsive element binding protein 1	2.02	0.009	1.67	0.009
*SELENBP1*	Selenium binding protein 1	−4.53	0.049	−1.07	0.89
*HBQ1*	Hemoglobin, theta 1	−2.62	0.048	1.14	0.71
*PTGDS*	Prostaglandin D2 synthase	−2.013	0.006	3.40	0.023

### Gene network analysis

**Figure 2:**
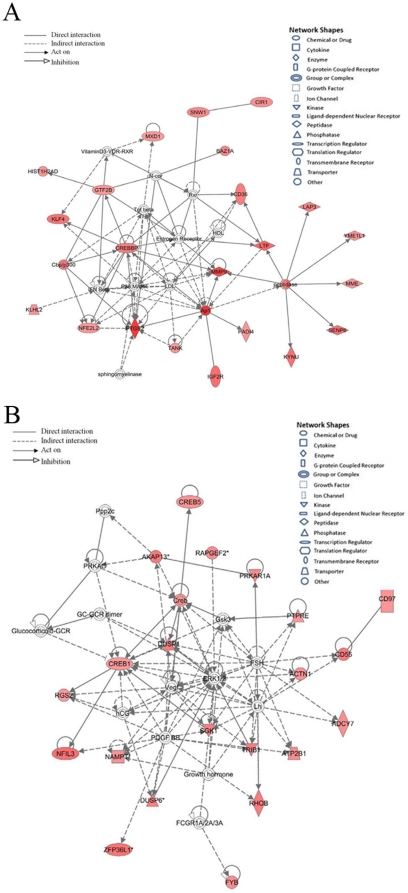
Most significant two gene networks of over-expressed genes in GBS patients. (**A**) Gene network involved in cardiovascular disease, hematological disease, neurological Disease; (**B**) gene network involved in amino acid metabolism, post-translational modification, small molecule biochemistry. Red: up-regulated in GBS compared to control. The intensity of the node color indicated the degree of up-regulation. Genes in uncolored notes were not identified as differentially expressed in our experiment and were integrated into the computationally generated networks on the basis of the evidence stored in the IPA knowledge memory indicating a relevance to this network.

To determine significant biological functions and to reveal transcriptional correlations among genes associated with GBS, the 256 significant genes were subjected to gene network analysis. The most significant disease and disorder biological functions associated with GBS-correlated genes were inflammatory response, infectious disease, and respiratory disease (Table 3 and Table S3). Cell death, cellular development and cellular movement were the top significant molecular and cellular functional categories. Hematological system development and function, immune cell trafficking and organismal survival were the most significant categories in physiological development and system function. Eighteen significant gene networks were noted in GBS (Table 4 and Table S4). *MMP9*, *PTGS2*, and *CREB1* were the ‘hub’ genes in the two top significant gene networks (Fig. 2A–B).

**Table 3:** Biological functions associated with GBS.

Network	Top Functions	*P* value	Focus genes
Disease and disorder			
1	Inflammatory response	1.01E-10 - 9.55E-03	57
2	Infectious disease	1.39E-08 - 1.12E-02	52
3	Respiratory disease	7.71E-07 - 7.50E-03	26
4	Cardiovascular disease	1.71E-06 - 1.11E-02	41
5	Organismal injury and abnormalities	1.71E-06 - 9.48E-03	23
Molecular and Cellular functions			
1	Cell death	2.32E-12 - 1.13E-02	79
2	Cellular development	8.26E-09 - 1.11E-02	64
3	Cellular movement	5.59E-08 - 1.11E-02	54
4	Cellular death and proliferation	5.45E-07 - 6.32E-03	75
5	Amino acid metabolism	1.32E-06 - 5.23E-03	25
Physiological system development and function			
1	Hematological system development and function	5.45E-07 - 1.11E-02	61
2	Immune cell trafficking	1.79E-06 - 8.60E-03	39
3	Organismal survival	4.12E-06 - 7.06E-03	37
4	Hematopoiesis	9.30E-06 - 1.11E-02	36
5	Tissue morphology	9.82E-06 - 6.34E-03	31

**Table 4:** List of the genes in most significantly up-regulated top five networks.

Network	Top functions	Score	Focus genes	Up-regulated genes in network
1	Cardiovascular disease, Hematological disease, Neurological disease	37	22	*BAZ1A, CD36, CIR1, CREBBP, GTF2B, HIST1H2AD, IGF2R, KLF4, KLHL2, KYNU, LAP3, LTF, MME, MMP9, MXD1, NFE2L2, PADI4, PTGS2, SENP6, SNW1, TANK, YME1L1*
2	Amino acid metabolism, Post-translational modification, Small molecule biochemistry	34	21	*ACTN1, ADCY7, AKAP13, ATP2B1, CD55, CD97, CREB1, CREB5, DUSP1, DUSP6, FYB, NAMPT, NFIL3, PRKAR1A, PTPRE, RAPGEF2, RGS2, RHOB, SGK1, TRIB1, ZFP36L1*
3	Inflammatory response, Antigen presentation, Cellular movement	34	21	*AIM2, C5AR1, CAMP, CASP1, CD163, CD1D, CSTA, DEFA3, FPR1, FPR2, G0S2, GBP2, GNAI3, IRAK3, IRF2, LY96, MCL1, NOD2, TLR1, TLR8, TNFAIP6*
4	Cell-to-cell signalling and interaction, Hematological system development and function, Hematopoiesis	33	20	*ANXA1, CD58, CRISPLD2, DPYSL2, HMGB2, HNRNPA2B1, HSPA6, KCTD12, MAP3K7, MARCKS, PICALM, PRKCB, PTGDS, RBM5, SP100, SRPK1, STXBP3, SUB1, TAOK3, ZMYND8*
5	Cellular development, Hematological system development and function, Hematopoiesis	24	16	*ACSL1, ARHGAP26, ATG3, ATG12, FOS, HHEX, IL10RB, ITGAM, LIMK2, MAFB, PLSCR1, PRKCD, RB1CC1, SELENBP1, SNX2, TSC22D1*

### Canonical pathway analysis

To gain further insights into the pathogenesis of GBS, we analyzed the GBS-correlated genes to elucidate dominant canonical pathways. 246 up-regulated and 10-down-regulated GBS correlated transcripts were subjected to canonical pathway analysis, which showed that 101 significant pathways in the up-regulated GBS gene set (Table S5). GnRH, Corticotropin releasing hormone and ERK/MAPK signaling pathways were the most significant pathways in the up-regulated GBS gene set (Table 5). Only two pathways, including Eicosanoid signaling and Pyruvate metabolism, were significant in the down-regulated GBS gene set (Table 6).

**Table 5:** List of the genes in most significantly up-regulated top ten canonical pathways.

Pathways	−log (*P* value)	Genes
GnRH signaling	8.15E00	*RAF1, PAK2, CDC42, CREBBP, CREB5, GNAI3, FOS, MAP3K7, PRKCD, CREB1, ADCY7, PRKAR1A, PRKCB*
Corticotropin releasing hormone signaling	5.71E00	*GNAI3, RAF1, FOS, PRKCD, CREB1, PTGS2, CREB5, ADCY7, PRKAR1A, PRKCB*
ERK/MAPK signaling	5.43E00	*RAF1, FOS, MAPKSP1, PAK2, YWHAH, DUSP1, PRKCD, DUSP6, CREB1, CREB5, PRKAR1A, PRKCB*
cAMP-mediated signaling	5.21E00	*RAF1, AKAP13, RGS2, MAPKSP1, DUSP1, DUSP6, CREB1, TDP2, CREB5, ADCY7, PRKAR1A*
Molecular mechanisms of cancer	5.06E00	*RAF1, PAK2, CDC42, CREBBP, JAK2, NBN, GNAI3, FOS, MAPKSP1, RHOB, MAP3K7, PRKCD, CFLAR, ADCY7, PRKCB, PRKAR1A*
IL-8 signalling	4.88E00	*GNAI3, RAF1, ITGAM, PAK2, RHOB, PRKCD, LIMK2, PTGS2, IRAK3, MMP9, PRKCB*
P2Y purigenic receptor signalling	4.64E00	*GNAI3, RAF1, FOS, PRKCD, CREB1, CREB5, ADCY7, PRKAR1A, PRKCB*
Toll-like receptor signaling	4.44E00	*FOS, TLR1, LY96, MAP3K7, TLR8 (includes EG:51311), IRAK3*
LPS-stimulated MAPK signalling	4.41E00	*RAF1, FOS, CDC42, MAP3K7, PRKCD, CREB1, PRKCB*
Renin-angiotensin signaling	4.15E00	*RAF1, FOS, PAK2, PRKCD, JAK2, ADCY7, PRKAR1A, PRKCB*

**Table 6:** List of the genes in all significantly down-regulated canonical pathways.

Pathways	−log (*P* value)	Genes
Eicosanoid signalling	1.42E00	*PTGDS*
Pyruvate metabolism	1.32E00	*HAGH*

**Figure 3:**
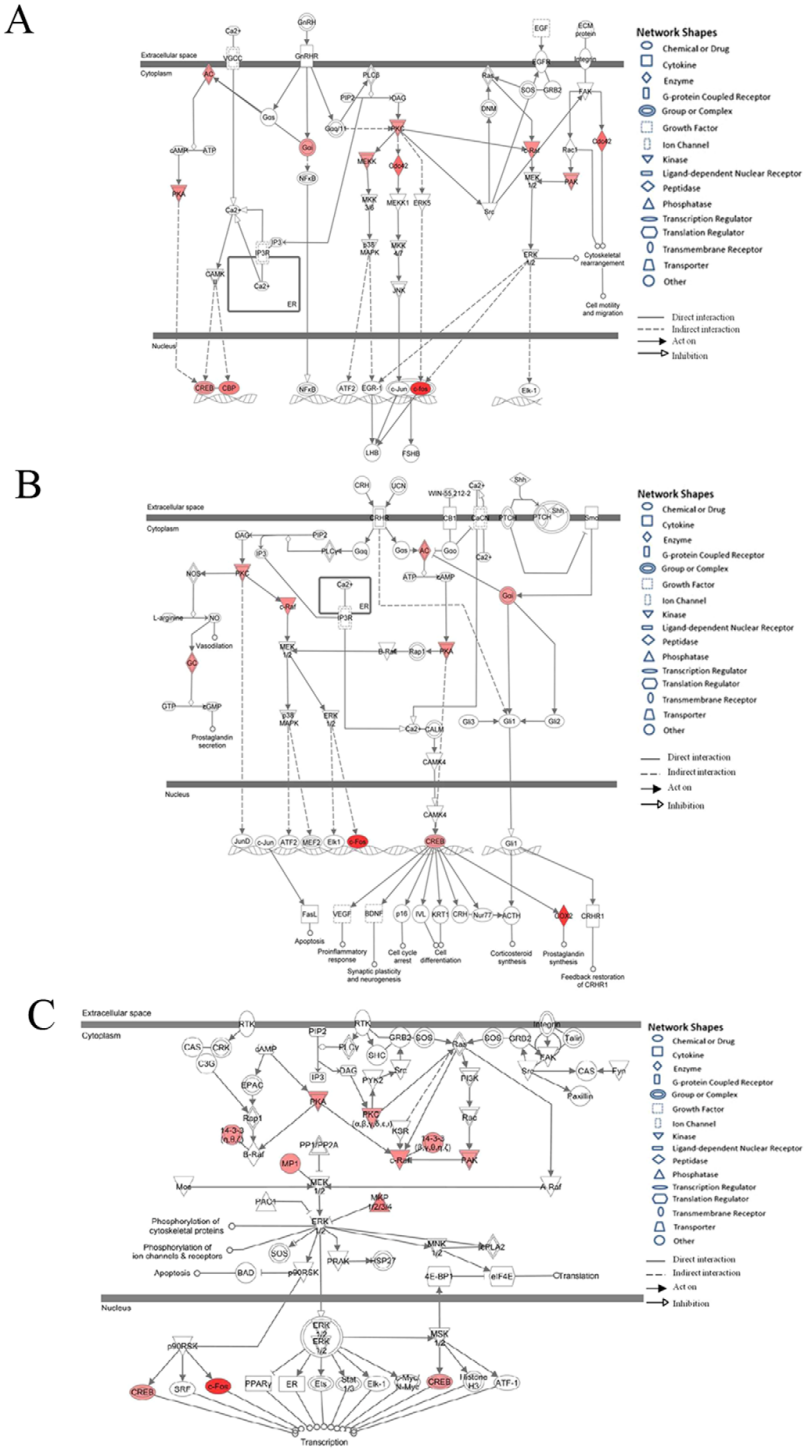
Most significant three canonical pathways of over-expressed genes in GBS patients. (**A**) GnRH signalling; (**B**) corticotropin releasing hormone signalling; (**C**) ERK/MAPK signalling. Red: up-regulated in GBS compared to control. The intensity of the node color indicated the degree of up-regulation. Genes in uncolored notes were not identified as differentially expressed in our experiment and were integrated into the computationally generated networks on the basis of the evidence stored in the IPA knowledge memory indicating a relevance to this pathway.

To demonstrate the biological interactions of these genes within these pathways and highlight hub genes controlling the signaling transduction, the top three up-regulated pathways are shown in Fig. 3.

## Discussion

In this study, we analyzed global gene expression of peripheral blood leukocytes in a clinically well-characterized and ethnically homogeneous cohort of GBS, and found several novel or reported candidate gene markers associated with the disease. Using gene networks and pathways analyses, we confirmed a likely role of several previously described biological processes and uncovered new important pathways that may be involved in the pathogenesis of GBS.

There were several interesting genes in our study that showed strong evidence of up-regulation, such as *FOS*, *PTGS2*, *HMGB2*, *MMP9*, *LY96*, *TTRAP*, *ANXA3* and *CREB1*. Among them, *FOS*, *PTGS2*, *HMGB2*, *LY96*, *TTRAP*, *ANXA3* and *CREB1* have never been reported to be associated with GBS. *FOS* gene encodes a transcription factor that has critical functions in regulating cell proliferation, differentiation, and transformation. The binding of FOS and JUN forms a dimeric transcription factor complex, activator protein-1 (AP-1). AP-1 affects the severity of inflammation by activation of cytokine production in cooperation with NFAT transcription factors and regulates the expression of IL-2, IL-3, GM-CSF, IL-4, IL-5, IL-13, IFN-gamma, TNF-alpha, CD40L, CD5, CD25, and IL-8 [37]. Therefore, *FOS* represents a GBS candidate gene for exploring the pathogenesis and also for a potential therapeutic target.

The protein encoded by *PTGS2* is a member of cyclo-oxygenase [38] family, a rate limiting enzyme catalyzing the synthesis of prostaglandins from arachidonic acid. It has been shown that a significant up-regulation of *PTGS2* was detected in sural nerves from patients with GBS and other demyelinating polyneuropathies [39]. In experimental autoimmune neuritis (EAN), an animal model for GBS, the administration of COX inhibitors significantly decreased clinical, neurophysiologic, and histomorphologic signs of the disease, indicating that COX and prostaglandins represent important factors in the regulation of the inflammatory demyelination of the peripheral nerves [40], [41], [42].

*HMGB2* encodes a member of the non-histone chromosomal high mobility group protein family and is associated with chromosomes during mitosis. Although the association of *HMGB2* and inflammation remains unclear, a closely related gene, *HMGB1*, has been demonstrated to exhibit an important extracellular function in mediation of inflammation processes [43].

*MMP9* is involved in the breakdown of extracellular matrix in normal physiological processes [44]. MMP9 may degrade myelin basic protein, one of the principal myelin components of the peripheral nervous system [45]. Similar to this report, it has been shown that elevated serum level of MMP9 was associated with disease severity and electrophysiological changes in GBS patients [18], [46], [47]. MMP9 expression can be detected in the damaged nerve of patients with GBS [48]. MMP9 has also been implicated in the pathogenesis of EAN [49], [50]. In particular, MMP9 is increased early in the course of EAN, peaking with maximum disease severity, and detected in nerve tissue in Schwann cells, endoneurial vessels, and infiltrating immune cells [49], [50]. The administration of an MMP inhibitor decreased severity of EAN [50], [51]. Thus, the inhibition of MMP9 could be a potential therapeutic strategy for GBS.

*LY96* is a small secreted glycoprotein that binds with cytokine-like affinities to both the hydrophobic portion of lipopolysaccharide and to the extracellular domain of TLR4 [52], which plays a critical role in Campylobacter jejuni-induced dendritic cell activation and B cell proliferation [11]. TLR4/LY96 complex is specific for recognition of lipopolysaccharide and promotes phagocytosis [52], [53]. In addition to inducing innate immune responses to microbial membrane components, TLR4/LY96 may sense tissue damage by responding endogenous ligands released from damaged tissues and induce inflammation [54]. Thus the elevation of LY96 is probably an indicator of inflammatory process.

*TTRAP* is reported to interact with members of the tumor necrosis factor receptor superfamily and may inhibit inflammation by inhibition of NFkB [55], [56]. The role of the up-regulation of *TTRAP* in GBS or other neuroimmunological diseases remains to be clarified.

*ANXA3* encoded a calcium-dependent phospholipid-binding protein that belongs to the annexin family [57]. The function of ANXA3 is yet to be fully elucidated. It has been suggested that ANXA3 expression is increased in post-ischemic brain [58]. In addition, ANXA3 also plays an important role in angiogenesis and neural tissue regeneration [58], [59]. In this study, *ANXA3* expression level is significantly correlated with the clinical severity in GBS, suggesting that ANXA3 may be used as a potential marker for prognostic monitoring in GBS patients.

The protein encoded by *CREB1* appears to regulate gene expression by constitutively binding to conserved cAMP-responsive elements [60]. Its pivotal role in gene networks has been revealed by bioinformatic analysis, which has estimated that there are approximately 4000 human genes containing conserved cAMP-responsive elements adjacent to the transcription start site [61]. Activation of CREB1 by phosphorylation has been shown to up-regulate the expression of *IL-2* and *IL-6* [62], [63], and to induce the transcriptional activation of *PTGS2* [64], whereby playing a critical role in inflammatory diseases.

Beyond the identification of individual genes, our analysis also focused on the identification and characterization of biological functions associated with these genes. The most significant biological functions involving genes with significantly altered expression included inflammatory response, infectious disease, cell death, cellular development, hematological system development and function, and immune cell trafficking. These data are consistent with findings of other studies revealing the altered cellular and immunological function in GBS [5], [65], [66], [67], [68], [69].

Although statistical significance of expression level changes may be one way to select a candidate gene for a given disease, gene network analysis offers the advantage of understanding the interaction of significant genes associated with a disease and the ability to find hub genes within a network that interact with several other genes up- and downstream of them. The high interconnectivity of hub genes with other correlated genes within a biological network may imply functional and biological importance of these genes. In this study, a number of hub genes of gene networks significantly associated with GBS, such as *CREB1*, *MMP9* and *PTGS*, have been identified. Regulating the expression of these hub genes could be important in the treatment of GBS.

The most significant canonical pathways involving genes with significantly altered expression included GnRH, corticotrophin releasing hormone and ERK/MAPK signaling. Extensive investigations suggest that the immune system may also modulate the hypothalamic-pituitary-gonadal and hypothalamic-pituitary-adrenal axis [70]. Generally, an increased immune response is coupled with an enhanced hypothalamic-pituitary-adrenal axis [71]. The up-regulation of GnRH and corticotrophin-releasing hormone signaling in GBS leukocytes may be a response in the immune system of patients affected by autoimmune diseases.

In addition to its crucial role in the production of pro-inflammatory cytokines [72], ERK/MAPK signaling is also involved in the demyelination process [73], [74], [75], [76]. Selective activation of ERK/MAPK signaling or alternatively overexpression of *RAF*, a molecule effector upstream of ERK1/2, prevents Schwann cell differentiation [74], [75]. *RAF* also induces demyelination of Schwann cell [75]. Furthermore, the blockage of ERK/MAPK signaling can rescue the demyelination caused by sustained activation of ERK/MAPK signaling [75]. Thus blockade of ERK/MAPK signaling could potentially inhibit both the inflammatory and demyelination processes, serving as a novel therapeutic target for GBS.

In the down-regulated gene set, Eicosanoid signaling and Pyruvate metabolism pathways were significantly involved. However, due to the paucity of gene hits, the alterations of these pathways need to be validated further.

In summary, this is the first report applying gene transcription analysis in the search for potential gene markers, studying gene biological functions and canonical pathways involved in GBS. As MMP9 has been shown in the damaged nerves of patients with GBS, and MMP9 expression in leucocytes is correlated to the clinical disability score, the level of peripheral nerve damages can be reflected by the changes in peripheral leukocytes. While the identification of reported GBS-associated genes *MMP9* authenticates this study, the discovery of novel candidate genes and the application of gene networks analysis in these markers highlight the transcriptional relationships among GBS-associated genes. It should be kept in mind that there are certain limits to *in silico* analysis. The small size of samples constrains the detection power in microarray. Since there are many undetermined gene-gene interactions, the actual relationship between genes may not be accurately revealed by the literature-based computational network. Despite these limitations, this is the first study describing a large number of GBS-associated genes in inflammatory cells. Further investigations are needed to confirm the clinical relevance of these biomarkers, and clarify the potential of ERK/MAPK signaling pathways as therapeutic targets in GBS disease models.

## Materials and Methods

### Ethics statement

This study was performed under a protocol approved by the Institutional Review Boards of Chang Gung Memorial Hospital (ethical license No: 96-0285B) and all examinations were performed after obtaining written informed consents.

### Study population

All the patients and controls were residents of Taiwan. Patient group consisted of GBS patients fulfilling the required diagnostic criteria [77]. None of the patients or the controls had systemic infection, autoimmune diseases, malignancies, or chronic renal, cardiac, or liver dysfunction.

### Sample collection

Venous puncture was performed between 1 and 2 weeks after onset of disease. The blood was collected into Paxgene™ blood RNA tube (Pre-AnalytiX, Qiagen). Total RNA of leukocytes was extracted using the Paxgene™ blood RNA Extraction Kit (Pre-AnalytiX, Qiagen), and transferred into the RNeasy MinElute spin column (RNeasy® MinElute®Cleanup Kit, Qiagen) for RNA purification and concentration. RNA quality was determined was determined using the A260/A280 absorption ratio and capillary electrophoresis on an Agilent 2100 Bioanalyzer automated analysis system (Agilent).

### Microarray mRNA expression profiling analysis

Genome-wide mRNA expression data of peripheral blood leukocytes in 7 treatment-naïve GBS patients (3 females and 4 males, age of onset: 52.43±15.06 years, mean score of GBS disability scale: 2.57±0.90, preceding infectious event: 1) and 7 healthy volunteers (3 females and 4 males, mean age: 50.00±14.06 years) were determined by Affymetrix Human Genome U133 plus 2.0 Arrays. All the samples from the patients with GBS were obtained within one month after disease onset. Biotin-labelled cRNA was generated and linearly amplified from 5 µg total RNA using the GeneChip® IVT Labeling Kit (Affymetrix) as described by the protocol. Array hybridization, chemiluminescence detection, image acquisition and analysis were performed using Partek >Genomics Suite following the manufacturer's instructions. Briefly, each microarray was first pre-hybridized at 55°C for 1 h in hybridization buffer with blocking reagent. Twenty µg biotin-labeled cRNA targets were first fragmented, mixed with internal control target and hybridized to the prehybridized microarrays in a volume of 1.5 ml at 55°C for 18 h. After hybridization, the arrays were washed with hybridization wash buffer and chemiluminescence rinse buffer. Enhanced chemiluminescent signals were generated by incubating arrays with alkaline phosphatase conjugated anti-digoxigenin antibody followed by incubation with Chemiluminescence Enhancing Solution and a final addition of Chemiluminescence Substrate. Images were collected for each microarray using the Affymetrix GeneChip® Scanner and the chemiluminescent signals were quantified, corrected for background and spot size, and spatially normalized. Obtained data were imported into GeneSpring GX 11.01 software for analysis (Agilent). The fold changes were analyzed by filtering the dataset using *P* value<0.05, two tailed Student's *t*-test. Additional filtering (minimum 2-fold change) was applied to identify the disease-related genes, which were analyzed using Ingenuity Pathway Analysis (IPA) software (Ingenuity Systems). Those genes with known gene symbols and their expression values were uploaded into the software. Each gene symbol was mapped to its own gene object in the Ingenuity Pathways Knowledge Database. Networks of these genes were assigned a score based on their connectivity. The score reflected the number of focus genes in the network and how relevant this network is to the original list of focus genes. A network graph was shown to present the molecular relationship between individual genes. The significance of the association between the data set and the canonical pathway was determined by a *P* value calculated using Fisher's exact test. *P*<0.05 was considered statistically significant. Microarray data are MIAME compliant and the raw data have been deposited with the NCBI Gene Expression Omnibus (http://www.ncbi.nlm.nih.gov/geo) under accession number GSE31014.

### Real-time polymerase chain reaction (RT-PCR)

Total RNAs were collected from the peripheral blood leukocytes of 16 treatment-naïve GBS patients (6 females and 10 males, age of onset: 47.06±15.28 years, mean score of GBS disability scale: 2.75±1.39, preceding infectious event: 3) within one month after disease onset and 20 healthy volunteers (10 females and 10 males, mean age: 51.85±11.25 years). RNA was converted to cDNA using the SuperScript® III First-Strand (Invitrogen). PCR results were generated using the 5′-nuclease assay (TaqMan) and the ABI 7900HT Sequence Detection System (Applied Biosystems). Each reaction included cDNA from 100 ng of RNA, 900 nM of each primer and 100 nM of probe and Universal PCR Master Mix (Applied Biosystems). Assay sequence information is indicated in Table S1. PCR parameters were 50°C for 2 min, 95°C for 10 min, 40 cycles of 95°C for 15 sec, 60°C for 1 min. Each sample was assessed in duplicate. Relative expression values were normalized to *β-actin*. Relative gene expressions were calculated using the 2^ΔCT^ method, ΔC_T_ = C_T_ (*β-actin*)−C_T_ (*target gene*), in which C_T_ indicates cycle threshold (the fractional cycle number where the fluorescent signal reaches detection threshold). Student's *t*- test was used to compare the differences between GPS and control groups. The correlation between gene expression level and GBS disability scale score was assessed by linear regression analysis.

### Enzyme-linked immunosorbant assay (ELISA)

Serum from the above population groups was collected for RT-PCR analysis. The serum level of MMP9 was assessed with a Quantikine ELISA kit (R&D System) according to the manufacturer's instruction. Student's *t*- test was used to compare the differences between GBS and control groups. The correlation between serum level of MMP9 and GBS disability scale score was assessed by linear regression analysis.

## Supporting Information

Table S1 **Lists of assay ID and probe sequence for RT-PCR.**
(DOC)

Table S2 **Lists of all up- or down-regulated genes in the leukocytes of GBS patients.**
(XLS)

Table S3 **Lists of significant disease and disorder biological functions associated with GBS-correlated genes.**
(XLS)

Table S4 **Lists of significant gene networks in GBS patients.**
(XLS)

Table S5 **Lists of significant pathways in the up-regulated GBS gene sets.**
(XLS)
