# Supplementary material for: Identification of Gene Networks and Pathways Associated with Guillain-Barré Syndrome
Source: PLoS One. 2012 Jan 10;7(1):e29506. doi: 10.1371/journal.pone.0029506 (PMC3254618; doi:10.1371/journal.pone.0029506)
Supplement: Table S1 — Lists of assay ID and probe sequence for RT-PCR. (DOC) [file pone.0029506.s001.doc]

**Supplementary Table 1** Lists of assay ID and probe sequence for RT-PCR

| Gene | Assay ID | Probe sequence |
| --- | --- | --- |
| ACTB | Hs99999903_m1 | CCTTTGCCGATCCGCCGCCCGTCCA |
| ANXA3 | Hs00971411_m1 | ACTTACTGTTGGCCATAGTTAATTG |
| CASP1 | Hs00354836_m1 | AATTTTCCGCAAGGTTCGATTTTCA |
| CREB1 | Hs00231713_m1 | TTCCTACAGGAAAATTTTGAATGAC |
| CREBBP | Hs00231733_m1 | TCAGTGCTAAGAGGCTGCAGACCAC |
| DEFA3 | Hs00414018_m1 | CTCCAAAGCATCCAGGCTCAAGGAA |
| FOS | Hs00170630_m1 | GTCAACGCGCAGGACTTCTGCACGG |
| HBQ1 | Hs00362218_g1 | CCAGCTTCCAGCTCCTGGGCCACTG |
| HMGB2 | Hs01127828_g1 | AAATATGAAAAGGATATTGCTGCAT |
| LTF | Hs00914334_m1 | CTCTGGTGCCTTCAAGTGTCTGAGA |
| LY96 | Hs00209770_m1 | CACCTACTGTGATAAAATGCAATAC |
| MMP9 | Hs00957562_m1 | CTCTATGGTCCTCGCCCTGAACCTG |
| PTGDS | Hs00168748_m1 | TCCAGCAGGACAAGTTCCTGGGGCG |
| PTGS2 | Hs00153133_m1 | GCTGGGCCATGGGGTGGACTTAAAT |
| SELENBP1 | Hs00259932_m1 | GGCAGCATGGCTACGAAATGTGGGA |
| TTRAP | Hs01099017_m1 | CCCAAGACCTATGTTGACCTAACCA |
